# Pyrrolidine Dithiocarbamate Might Mitigate Radiation-Induced Heart Damage at an Early Stage in Rats

**DOI:** 10.3389/fphar.2022.832045

**Published:** 2022-03-22

**Authors:** Yajing Wu, Lina Liu, Shengliang Lv, Yi Wang, Shuai Wang, Sheng Wang, Jiandong Zhang, Jun Wang

**Affiliations:** ^1^ Department of Radiation Oncology, Fourth Hospital of Hebei Medical University, Shijiazhuang, China; ^2^ Hebei Key Laboratory of Neurophysiology, Shijiazhuang, China; ^3^ Department of Radiation Oncology, The First Affiliated Hospital of Shandong First Medical University, Jinan, China

**Keywords:** acute radiation-induced heart pro-fibrotic damage, left ventricular function, nuclear factor-kappa B, pyrrolidine dithiocarbamate, hypoxia-inducible factor-1α, connective tissue growth factor

## Abstract

**Objective:** Radiation-induced heart damage (RIHD) is becoming an increasing concern due to offsetting clinical benefits of radiotherapy to a certain extent. Pyrrolidine dithiocarbamate (PDTC) as an antioxidant has been implicated in cardioprotective effects. We aimed to investigate whether pyrrolidine dithiocarbamate could attenuate heart damage at an early stage post-irradiation and unveil the potential mechanisms.

**Methods**: A total of 15 adult male Sprague–Dawley rats were randomized into the control, irradiation (IR), and PDTC plus irradiation (PDTC + IR) groups. Hearts were irradiated with a single fraction of 20.0 Gy. Rats received daily intraperitoneal injection of PDTC for 14 days. At the 14th day post-irradiation, echocardiography was performed, and rats were killed. Morphological damage was examined by hematoxylin–eosin (HE) stain and Masson’s trichrome stain. The collagen volume fraction (CVF) was applied for semi-quantitative analysis. The protein levels were analyzed by Western blot and mRNA levels by quantitative real-time PCR.

**Results**: No significant damage to systolic function of left ventricular was induced at an early stage post-irradiation. HE staining of cardiac tissue showed that the disordered arrangement of myocardial cells and abnormal cell infiltration were alleviated in the PDTC + IR group. The increased CVF in the irradiation group was inhibited in the PDTC + IR group (22.05 ± 2.64% vs. 9.99 ± 1.65%, *p* < 0.05). The protein levels of nuclear factor-kappa B (NF-κB), hypoxia-inducible factor-1α (HIF-1α), and COL-1 were downregulated after treatment with PDTC (*p* < 0.05), and there was a declining trend in the protein of the connective tissue growth factor (CTGF). The mRNA expression of NF-κB and HIF-1α in the PDTC plus irradiation group was lower than that in the irradiation group (*p* < 0.05), and there was a declining trend in the mRNA expression of the connective tissue growth factor and COL-1.

**Conclusion:** PDTC alleviates myocardial cell disordered arrangement, abnormal cell infiltration, and pro-fibrotic change at an early stage in rats with radiation-induced heart damage. Such a protective effect is closely associated with the downregulation of NF-κB.

## 1 Introduction

A total of 2 million new cancer patients are diagnosed per year, and improvements in cancer therapies have increased cancer survivors (Survivorship, 20192019). Among these survivors, cardiovascular disease is the leading cause of non-cancer-related mortality (Mehta et al., 2018). Detrimental effects on the cardiovascular system related to cancer treatments are a clinical challenge for cardiologists and oncologists. Radiotherapy may cause damage to both acute and chronic epicardial coronary artery and microcirculatory as well as fibrotic changes in the valve or pericardium (Chang et al., 2017). Pericarditis and pericardial effusions are the early-onset side effects that develop within weeks, while others have a late onset, often 10–20 years after treatment such as valvular heart disease and heart failure (Hufnagle et al., 2021). The mechanisms of radiation-induced cardiac damage are complicated, multi-factorial, and under-recognized, initial inflammation or oxidative stress and subsequent fibrosis seem to be the potential events (Ping et al., 2020). Accumulating evidence generally indicates that the main pathological manifestation of radiation-induced heart damage (RIHD) is myocardial fibrosis, and that radiation-induced heart fibrosis is usually considered as chronic manifestation. So far there are no approaches to reverse RIHD (Boerma 2012; Gürses et al., 2014). The administration of a palladium lipoic acid complex (POLY-MVA) mitigated the damage of radiation to mitochondria. Nonetheless, POLY-MVA did not mitigate adverse cardiac remodeling at 18 weeks after rats were exposed to the local heart with X-rays (Sridharan et al., 2017). Therefore, it is necessary to explore the pathogenesis of RIHD and find an effective intervention against RIHD at an early stage.

Recently, we demonstrated that radiation-induced myocardial fibrotic change could be observed as early as 14 days after exposure to irradiation, and the mechanisms might be attributed to the activation of oxidative stress and endoplasmic reticulum stress (Wang et al., 2016). NF-κB is considered as a redox-sensitive transcription factor and a regulator of the inflammatory process (Han et al., 2013; Sies et al., 2017). The p65/50 heterodimer is a common pattern of NF-κB, which exists in the cytoplasm as an inactive component bound to the inhibitor of κB alpha (IκB α). Once activated, the protein of IκB is phosphorylated and ubiquitinated, subunits of the NF-κB including p65 and P50 are released. NF-κB classical pathway activation is mainly marked by the formation of P50 and P65. Studies have also showed that ionizing radiation could induce NF-κB activation (Meng et al., 2003), and in the experimental studies of tumor cells, the activation of the NF-κB pathway was also confirmed (Cataldi et al., 2003; Lam et al., 2015). However, evidence of the role of NF-κB in acute RIHD is lacking. Irradiation could induce a reduction in the myocardial micro-vessel density (Sridharan et al., 2017) and subsequent myocardial ischemia and hypoxia. NF-κB increased the HIF-1 mRNA expression as an important factor, and this was followed by transcription of some pro-inflammatory genes (Korbecki et al., 2021). Hypoxia-inducible factor-1α (HIF-1α) is also one of the classic factors which can lead to fibrosis and upregulates the expression of many fibrosis-related genes, such as the connective tissue growth factor (CTGF) and transforming growth factor-β1 (TGF-β1). CTGF can increase the synthesis of fibrosis-related factors, such as collagen type I, and accelerate the proliferation of fibroblast (Zhou et al., 2013). Whether NF-κB, HIF-1α, and CTGF were activated at the acute stage of RIHD and participated in the process of radiation-induced myocardial fibrosis remains unexplored.

PDTC, an oxidant and an inhibitor of NF-κB, could inhibit cardiac inflammatory response, relieve ventricular remodeling, maintain normal tissue structure, and protect cardiac function in the rat’s model with hypertension and myocardial ischemia (Theuer et al., 2002; Cau et al., 2015). There are very few reports about the effect of PDTC on RIHD, especially at an early stage post-irradiation. The most important finding in our previous data was that radiation-induced heart damage at a very early stage could be inhibited. We previously reported that chronic intermittent hypobaric hypoxia (CIHH) treatment prior to irradiation alleviated the early myocardial fibrosis by inhibiting oxidative stress and endoplasmic reticulum stress (Wang et al., 2016). Evidence of the protective effect of PDTC on RIHD is unavailable, and it is worth exploring whether PDTC could exert cardiac protection by the downregulation of NF-κB.

Here, we asked whether PDTC could alleviate RIHD in a rat model at an early stage and sought to investigate and delineate the putative cellular mechanism. Our data indicated that PDTC attenuated morphological damage and pro-fibrotic cardiac changes at an early stage in rat hearts subjected to irradiation, most likely *via* the inhibition of NF-κB, HIF-1α, and COL-1, providing a potentially clinical strategy to attenuate RIHD.

## 2 Materials and Methods

### 2.1 Animals

A total of 15 adult male Sprague–Dawley rats aged 10 weeks, weighed 200–250 g were obtained from the Experimental Animal Center of Hebei Medical University (Shijiazhuang, China), with Guide for the Care and Use of Laboratory Animals, and it was approved by the Animal Care and Ethical Committee of Hebei Medical University. All rats were randomized into three groups: control, irradiation, and PDTC plus irradiation. The program was approved by the Animal Care and Ethical Committee of Hebei Medical University. Animals were synchronized for a 12:12-h light–dark cycle (lights on at 8 am and lights off at 8 pm), housed individually, and allowed to move freely in standard plastic cages in a climate-controlled room (22 ± 1°C). Food and water were provided *ad libitum*.

### 2.2 Pyrrolidine Dithiocarbamate Treatment

**FIGURE 1:**
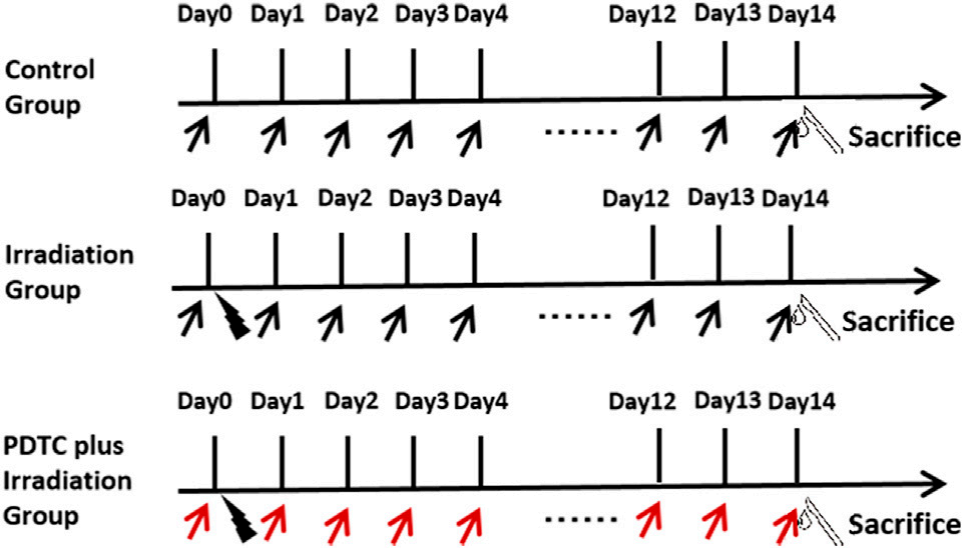
Schedule of the experiment. Hearts of rats were irradiated with a single fraction of 20.0 Gy (black lightning bolt). PDTC (red arrow) or normal saline (black arrow) was intraperitoneally administrated at the dosage of 120 mg/kg 30 min prior to irradiation and continued to receive the daily injection until the 14th day post-irradiation.

PDTC (p8765-5G, SIGMA, United States) was dissolved in physiological saline (0.9%) on the experiment day. In the PDTC plus irradiation group, rats received an intraperitoneal injection of PDTC at a dosage of 120 mg/kg 30 min prior to radiation and continued to receive the daily injection until the 14th day after radiation when cardiac tissue was harvested. Rats in control and irradiation groups were administrated with the corresponding volume of normal saline (Figure 1).

### 2.3 Irradiation treatment

Rats were anesthetized with 10% chloral hydrate (0.35 ml/100 g) and were irradiated with a single fraction of 20.0 Gy from a precise type medical high-energy linear accelerator (Elekta Corporation, Sweden) operated at 6 MV X-ray and with a dose rate of 1.87 Gy/min. Radiation was delivered locally to rat hearts using parallel opposed fields (anterior:posterior 1:1) with a diameter of 19 mm, while the rest of the rat body parts was shielded with lead plates (Figure 1).

### 2.4 Left Ventricular Function Measurement *In Vivo*

At the 14th day after irradiation, echocardiography was used for heart wall dimension measurements and to measure left ventricular ejection fraction (LVEF) by a well-trained investigator. The study measured the LV diastole and systole according to the main laws of the American Society of Echocardiography (Lang et al., 2015). Echocardiography was performed with a high-frequency transducer probe (VisualSonics MS400, FUJIFILM VisualSonics, Inc. Toronto, Canada, with a frequency range of 18–38 MHZ). Initially, the rats were anesthetized with 3% isofurane (80% oxygen) and placed supinely on an electrical heating pad (37°C). During examination, the isofurane concentration was reduced to a minimum (1–2%) to obtain constant and comparable heart rates shown by ECG. Additionally, prewarmed ultrasound gel was applied to reduce cold stimulation to small animals. Interventricular septum and para-sternal short-axis images were acquired. Conventional indicators were measured from the LV M-mode in short-axis view for three consecutive cardiac cycles and then averaged; these indicators included the LV wall thickness, diameter, fractional shortening (FS [LV end-diastolic diameter−LV end-systolic diameter]/LV end-diastolic diameter×100), and ejection fraction (EF [LV end-diastolic volume−LV end-systolic volume]/LV end-diastolic volume×100).

### 2.5 Histological Evaluations

#### 2.5.1 Hematoxylin–Eosin (HE) Staining

At the 14th day after irradiation, cardiac tissues were harvested, and morphological changes in myocardial cells and interstitial were observed *via* HE staining. Hearts were dissected rapidly from the mediastinum and placed in 10% buffered formaldehyde for 24 h. An average of 3- to 4-mm-thickness longitudinal tissue slices showing the four chambers of the heart were taken after following the routine tissue processing. Sections (5 μm thick) were stained using hematoxylin and eosin for general tissue characterization. The nucleus of a normal myocardial cell was stained blue, and the cytoplasm was stained red. Fibroblast cells were distributed among the myocardial cells. The nucleus of a normal fibroblast cell was stained dark blue, and the cytoplasm was stained red.

#### 2.5.2 Masson’s Trichrome Stain

Total collagen accumulation was determined by preparing tissue sections with Masson’s trichrome stain, and collagen volume fraction (CVF) was applied for semi-quantitative analysis of myocardial collagen (Wang et al., 2016). Briefly, the myocyte and collagen were stained red and green, respectively, with Masson’s trichrome staining. CVF was applied for semi-quantitative analysis of myocardial collagen *via* an image processing system (Motic Med 6.0, Xiamen, China). Interstitial CVF was calculated as the area occupied by the green-dyed tissue, divided by the total myocardial area under direct vision. For each animal, five microscopic fields were examined, and the average of CVF was computed.

### 2.6 Determination of Nuclear Factor-Kappa B, Hypoxia-Inducible Factor-1α, Connective Tissue Growth Factor, and COL-1 Protein *via* Western Blot

At the 14th day after irradiation, rat heart tissue was harvested, snap frozen, crushed in liquid nitrogen, and then weighed. The tissue powder was homogenized with ice-cold Roche buffer with protease inhibitors (300 μl), and then was centrifuged at 20,000 g for 15 min at 4°C. The supernatant was collected to detect the protein level by BCA assay and diluted to the same concentration by thoroughly mixing with lysis buffer and loading buffer based on the concentration determined. SDS-PAGE was performed on 10% gradient gels. Samples, each containing 20 μg of protein, were added in the same amount of the sample-loading buffer. After electrophoresis, proteins were transferred to a polyvinylidene difluoride membrane and were blocked with 5% skim milk TBST buffer. Blots were incubated with primary antibodies overnight at 4°C and incubated with horseradish peroxidase-conjugated secondary antibody (EarthOx, Millbrae, CA). The signal was detected using the Clinx chemiluminescence’s system (Shanghai, China). Primary antibodies P50 (ab32360, Abcam, United States), P65 (ab7970, Abcam, United States), HIF-1 alpha (sc-10790, SANTA CRUZ Biotech Corp, United States), CTGF (ab6992, Abcam, United States), COL-1 (COL1A antibody) (sc-59772, SANTA CRUZ Biotech Corp, United States), and beta-actin (Bioworld Technology Corporation) were used for Western blot analysis. The best concentration of primary antibody (P50:1:1,000; P65: 1:400; HIF-1 alpha: 1:100; CTGF: 1:1,000; COL-1: 1:400; and *β* -actin:1:5,000) were determined.

### 2.7 Detection of Nuclear Factor-Kappa B, Hypoxia-Inducible Factor-1α, Connective Tissue Growth Factor, and COL-1 mRNA *via* Quantitative Real-Time Polymerase Chain Reaction

On the 14th day after irradiation, to assess the mRNA levels of NF-κB and the downstream pathway, 50 mg of myocardial tissue were harvested, snap frozen, crushed in liquid nitrogen, and then weighed. Total RNA was extracted using an RNeasy Mini Kit (Qiagen, CA). RNA levels were quantified using 1 μl of each sample, and the process was repeated twice. The first strand cDNA was synthesized from 1 μg of each total RNA sample using reverse transcriptase. The temperature protocol for cDNA reverse transcription reactions was as follows: 25°C for 5 min, 50°C for 15 min, and 85°C for 5 min, and then the cDNA sample was frozen at −20°C. Primer Premier 5.0 (Premier Corporation, Canada) was used to design primers (sequences in Table 1). The primers were frozen at −20°C. PCR for each gene was performed with an Applied Biosystems QuantStudio 6 Flex fluorescent quantitative PCR instrument and SYBR Green I, and the amplification and melt curves were obtained. The thermal cycler protocol for all PCR reactions was as follows: 95°C for 2 min, 95°C for 15 s, and 60°C for 1 min for a total of 42 cycles. The 2^-△△CT^ method was used for relative quantitative analysis, and the difference among the samples was detected.

### 2.8 Statistical Analysis

Data were analyzed by Statistical Product and Service Solutions (SPSS) version 22.0 statistical software (IBM Co., Armonk, NY, United States). First, a normality test was conducted, and one way analysis of variance (ANOVA) was adopted for comparisons in three groups. The non-normality test was compared by using the rank sum test. When the homogeneity of variance was assumed, the LSD method was used for pairwise comparison, and if not, Dunnett’s C test was used for pairwise comparison. Data were expressed as mean ± standard error. The criterion for statistical significance was *p* < 0.05.

## 3 Results

### 3.1 No Significant Damage to Systolic Function of Left Ventricular Was Induced by Irradiation at a Very Early Stage

**FIGURE 2:**
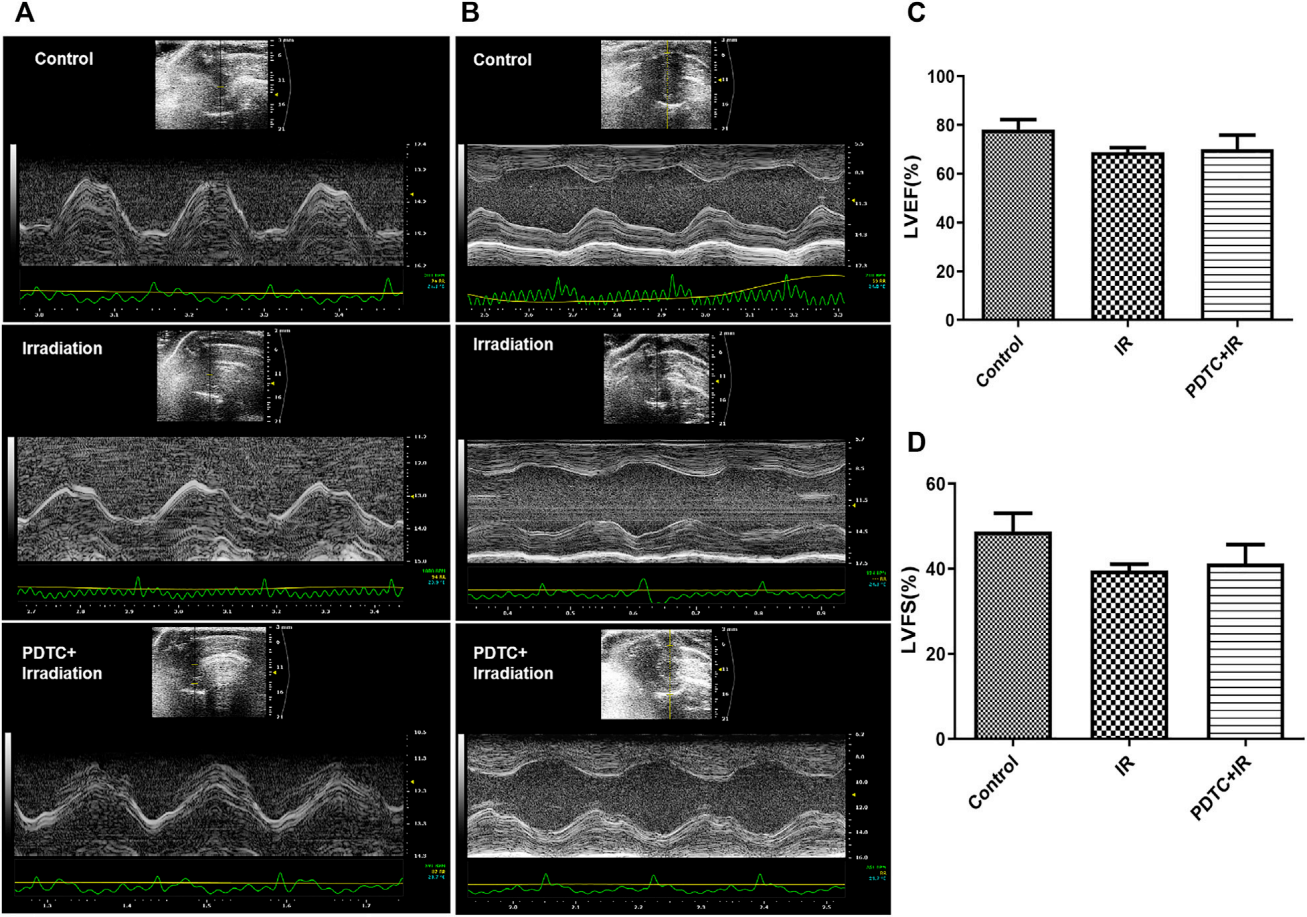
Electrocardiographs of the short axis view in M-mode in rat hearts. **(A)** Interventricular septum image. **(B)** Para-sternal short axis view. **(C)** LVEF of rats from each group. **(D)** LVFS of rats from each group.

The evaluation of left ventricular function was performed at the 14th day after the irradiation injury. Electrocardiograph of the short axis view of the M-mode in rat hearts was shown with the interventricular septum image (Figure 2A) and para-sternal short axis view (Figure 2B). LVEF and LVFS are commonly used to evaluate the cardiac systolic function. Both the parameters of LVEF and LVFS were lower in the irradiation group than that in the control group, but there was no significance among the groups (*p* > 0.05 for both, Figures 2C,D). In parallel, there was no significant difference of LVEF and LVFS between the irradiation and PDTC + irradiation groups. The results indicated that no abnormal impairment of left ventricular systolic function was detected at a very early stage in an RIHD rat model.

### 3.2 No Significant Structural Changes Was Induced by Irradiation in Echocardiography and Pyrrolidine Dithiocarbamate-Alleviated Radiation-Induced Myocardial Morphological Change

**FIGURE 3:**
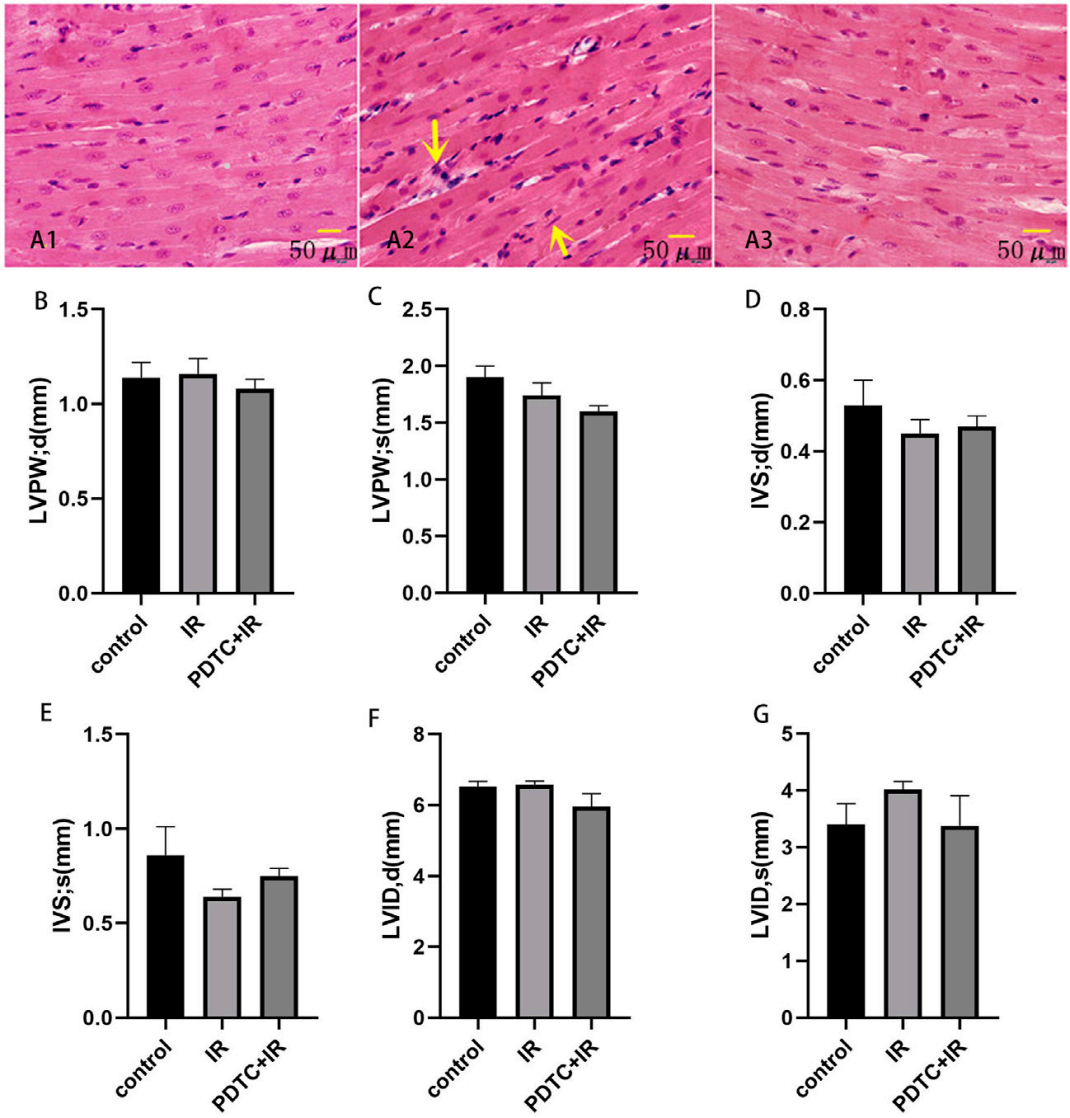
Effects of PDTC on myocarditis reaction (n = 5). Transverse section in the cardiac muscles of rats *via* HE staining for the observation of cardiac injury (×200). **(A1)**: control group; **(A2)**: irradiation group; **(A3)**: PDTC + irradiation group. Arrows were abnormal cells infiltration. **(B,C)** show the thickness of LVPW at diastole and systole. **(D,E)** show the thickness of IVS at diastole and systole. **(F,G)** show LVID at diastole and systole.

Images of the interventricular septum (Figure 2A) and para-sternal short axis (Figure 2B) were displayed with the M-mode. The left ventricle posterior wall (LVPW) thickness at diastole and systole, interventricular septal (IVS) thickness at diastole and systole, and left ventricular internal diameters (LVID) at diastole and systole were similar between the control and irradiation groups (Figure 3B–G).

HE staining was performed to observe the general morphological characters of myocardial tissue. Myocardial cell edema, arrangement disorder, pyknosis of myocardial nuclei, and abnormal cells infiltration could be observed after irradiation. However, the aforementioned damage was attenuated in the PDTC plus irradiation group compared with the irradiation group (Figure 3A).

### 3.3 Pyrrolidine Dithiocarbamate-Alleviated Radiation Induced Myocardial Pro-Fibrotic Change

**FIGURE 4:**
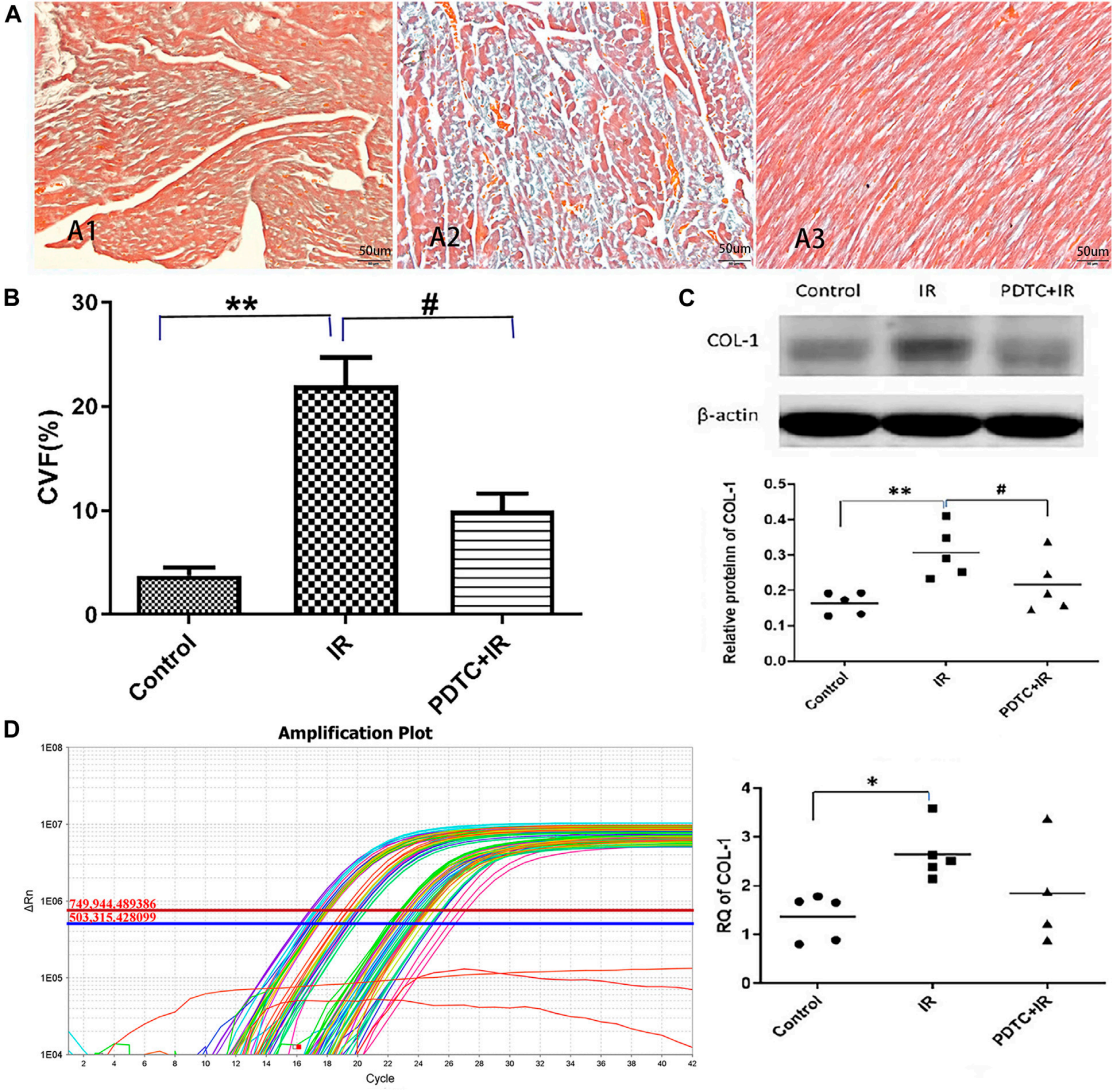
Effects of PDTC treatment on radiation-induced myocardial fibrosis. **(A)** Transverse section in the myocardial of rats *via* Masson’s trichrome staining for the observation of cardiac fibrosis (×200). All the collagen fibers were stained blue. **(A1)** Normal appearance in the control group. **(A2)** Damage characterized by myocardial interstitial fibrosis in the irradiation group. **(A3)** Little of collagen deposited at myocardial interstitial tissue in the PDTC + irradiation group. **(B)** Quantitative analysis showing collagen volume fraction (CVF) in each group. **(C)** Expression of COL-1 was revealed by Western blot in each group. **(D)** Expression of COL-1 was revealed by qPCR in each group. n = 5 for each group. *p < 0.05 for irradiation vs. control group; **p < 0.01 for irradiation vs. control group; #*p <* 0.05 for PDTC + irradiation vs. irradiation group.

Masson’s trichrome stain was performed to determine whether the pro-fibrotic change at a very early stage in rats subjected to irradiation. As shown in Figure 4A, collagen fibers in the irradiation group were more widely distributed in the myocardial interstitium than in the control group, but this increase in distribution was significantly attenuated in the PDTC plus irradiation group. The semi-quantitative analysis of Masson’s trichrome stain (Figure 4B) indicated that the collagen volume fraction (CVF) of the irradiation group was significantly higher than that of the control group (22.05 ± 2.64% vs. 3.76 ± 0.79%, *p* < 0.01, *n* = 5), but the enhancement was inhibited by PDTC treatment (9.99 ± 1.65% vs. 22.05 ± 2.64%, *p* < 0.05, *n* = 5).

Similar pattern could be observed in the COL-1 protein level as detected by Western blot, which is a marker of fibrosis. The level of COL-1 protein was increased in the irradiation group compared with the control group (0.52 ± 0.05 vs. 0.32 ± 0.04, *p* < 0.01, *n* = 5, Figure 4C), but the increase in COL-1 in the PDTC plus irradiation group was much smaller than that in the irradiation group (0.36 ± 0.03 vs. 0.52 ± 0.05, *p* < 0.05, *n* = 5, Figure 4C).

Furthermore, according to Figure 4D, it showed that the level of COL-1 mRNA in the irradiation group was higher than that in the control group (2.65 ± 0.25 vs. 1.26 ± 0.24, *p* < 0.05, *n* = 5), but the increase trend of COL-1 mRNA in the PDTC plus irradiation group was slightly smaller than that in the irradiation group (1.85 ± 0.55 vs. 2.65 ± 0.25, *0.05<p < 0.10*, *n* = 5).

### 3.4 Pyrrolidine Dithiocarbamate-Attenuated Radiation Induced Myocardial Inflammation and Downstream Pathway

#### 3.4.1 Effects of Pyrrolidine Dithiocarbamate on the Nuclear Factor-Kappa B Pathway

**FIGURE 5:**
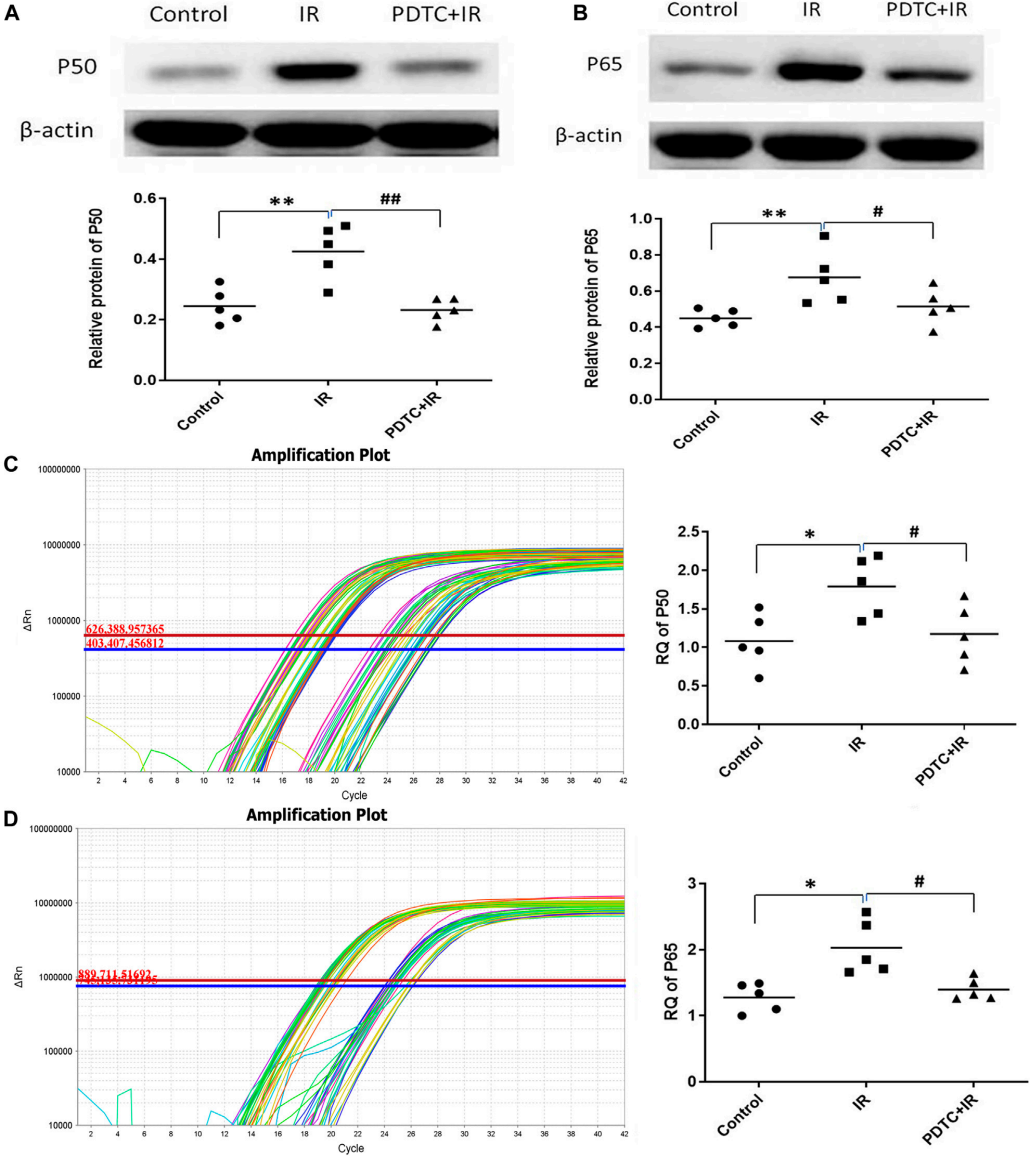
Effects of PDTC treatment on the expression of NF-κB protein and mRNA in the myocardium. **(A)** and **(B)** show the expression of P50 and P65 proteins revealed by Western blot. **(C)** and **(D)** show the mRNA levels of P50 and P65 analyzed by qPCR (*n* = 5). *p < 0.05 for irradiation vs. control group; **p < 0.01 for irradiation vs. control group; ^#^ *p <* 0.05 for PDTC + irradiation vs. irradiation group; ^##^ *p <* 0.05 for PDTC + irradiation vs. irradiation group.

NF-κB classical pathway activation is mainly marked by the formation of P50 and P65. The expression of P50 and P65, the two main proteins of activation of NF-κB, was analyzed using Western blot to address whether NF-κB as one of the inflammation master switch was activated by radiation. As shown in Figures 5A,B, the expression of P50 and P65 proteins in the irradiation group was significantly higher than that in the controls (0.43 ± 0.04 vs. 0.2 ± 0.03, *p* < 0.01 for P50; 0.68 ± 0.07 vs. 0.45 ± 0.02, *p* < 0.01 for P65, *n* = 5), suggesting the involvement of NF-κB activation in irradiation-induced heart damage. PDTC treatment inhibited the increase in the expression of both P50 and P65 induced by irradiation (0.23 ± 0.02 vs. 0.43 ± 0.04, *p* < 0.01 for P50; 0.51 ± 0.05 vs. 0.68 ± 0.07, *p* < 0.05 for P65, *n* = 5, Figures 5A,B).

Results of qPCR (Figures 5C,D) showed that mRNA levels of both P50 and P65 were significantly higher in the irradiation group than in the controls (1,79 ± 0.17 vs. 1.08 ± 0.16, *p* < 0.05 for P50; 1.98 ± 0.20 vs. 1.28 ± 0.10, *p* < 0.05 for P65, *n* = 5), but the increase in RQ was significantly decreased in the PDTC plus irradiation group (1.18 ± 0.17 vs. 1.79 ± 0.17, *p* < 0.05 for P50; 1.40 ± 0.07 vs. 1.98 ± 0.20, *p* < 0.05 for P65, *n* = 5), which was consistent with the results of Western blot.

#### 3.4.2 Effects of Pyrrolidine Dithiocarbamate on Hypoxia-Inducible Factor-1α and Connective Tissue Growth Factor

**FIGURE 6:**
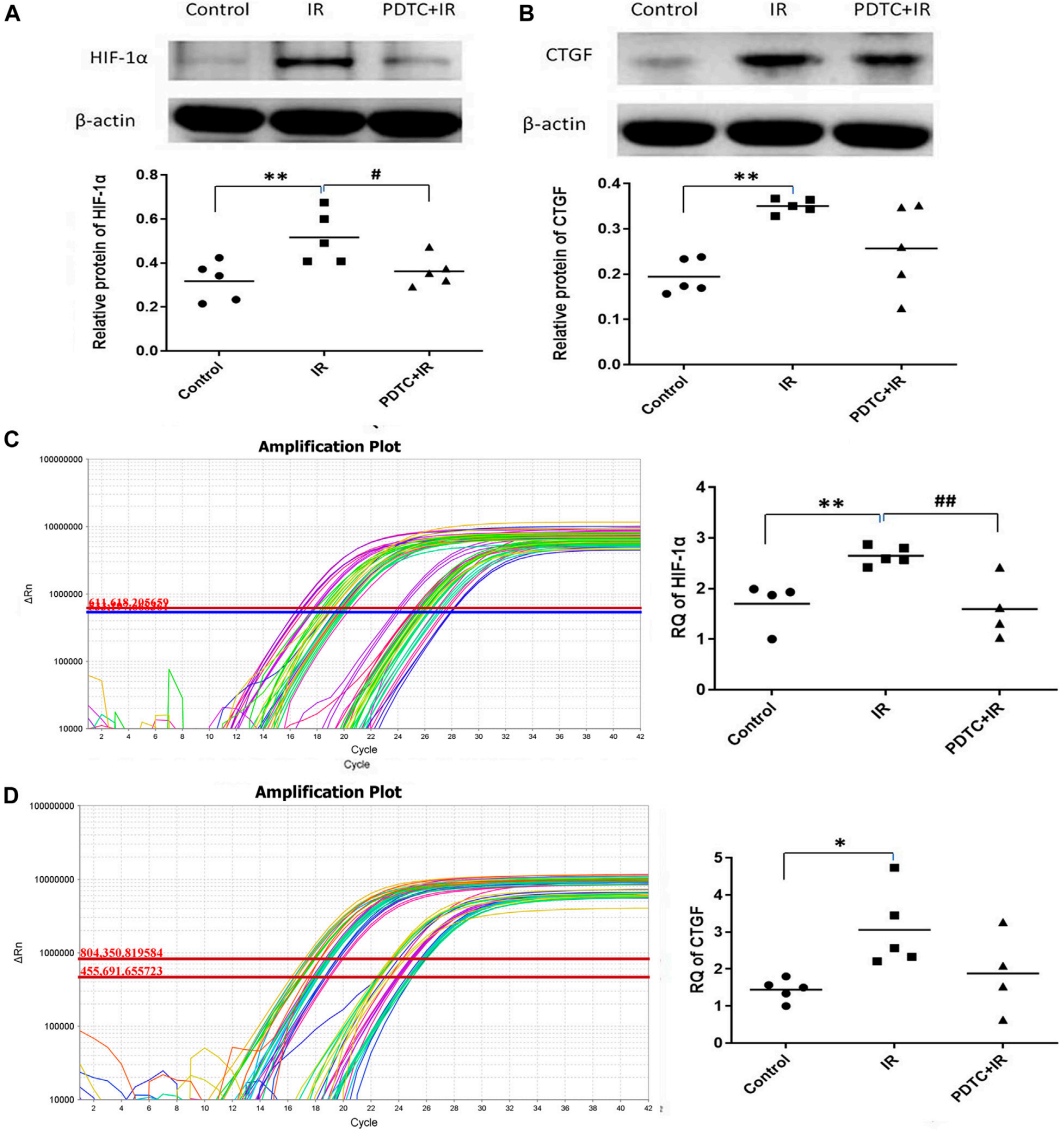
Effects of PDTC on the expression of HIF-1α and CTGF proteins and mRNAs in myocardial tissue. **(A)** and **(B)** show the HIF-1α and CTGF protein levels revealed by Western blot **(C)** and **(D)** show the mRNA levels of HIF-1α and CTGF analyzed by qPCR. *n* = 5. *p < 0.05 for irradiation vs. control group; **p < 0.01 for irradiation vs. control group; ^#^ *p <* 0.05 for PDTC + irradiation vs. irradiation group; ^##^ *p <* 0.05 for PDTC + irradiation vs. irradiation group.

Activated NF-κB increased the HIF-1 mRNA expression. HIF-1α might increase the expression of many fibrosis-related genes, such as CTGF. Therefore, it was hypothesized that the expression of HIF-1α and CTGF genes was regulated by the activated NF-κB, and they might serve as the intermediate connection to radiation-induced cardiac fibrosis. As shown in Figure 6A, the HIF-1α protein level in the irradiation group was significantly higher than that in the controls (0.31 ± 0.03 vs. 0.16 ± 0.01, *p* < 0.01, *n* = 5), but the enhancement was significantly inhibited by PDTC treatment (0.22 ± 0.04 vs. 0.31 ± 0.03, *p* < 0.05, *n* = 5). As shown in Figure 6B, the CTGF protein level in the irradiation group was significantly higher than that of the control group (0.52 + 0.05 vs. 0.32 ± 0.04, *p* < 0.01, *n* = 5), but the increase was slightly smaller in the PDTC plus irradiation group than that in the irradiation group (0.36 ± 0.03 vs. 0.52 + 0.05, 0.05*< p <* 0.10, *n* = 5).

Results of qPCR (Figure 6C) showed that HIF-1α mRNA was 2.65 ± 0.08 in the irradiation group, exhibiting an increase compared with 1.70 ± 0.23 in the control group (*p* < 0.01, *n* = 5), but the increase was dramatically inhibited by PDTC treatment (1.59 ± 0.30 vs. 2.65 ± 0.08, *p* < 0.01, *n* = 5). As shown in Figure 6D, the CTGF mRNA level in the irradiation group was 3.06 ± 0.47, a significant increase compared with 1.42 ± 0.17 of the control group (*p* < 0.05, n = 5); but the upward trend was slightly inhibited by PDTC treatment (1.88 + 0.55 vs. 3.06 ± 0.47, 0.05*<p* < 0.10, *n* = 5).

### 3.5 PPI Network Analysis

**FIGURE 7:**
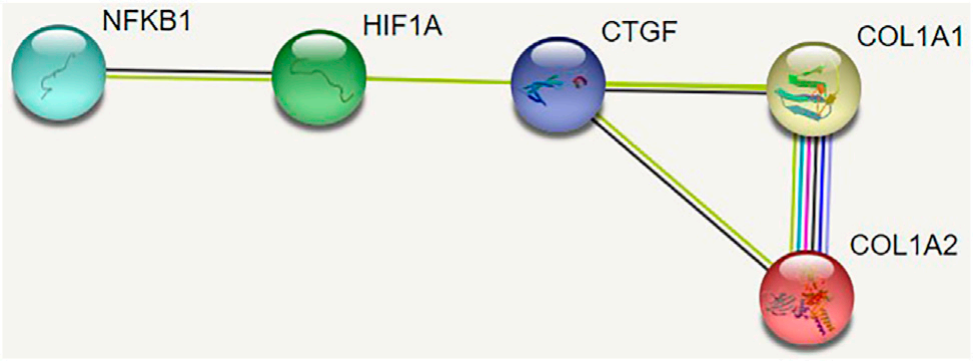
PPI network was used to analyze interactions between NF-κB, HIF-1α, CTGF, and COL-1A.

The PPI network was used to analyze the interactions among NF-κB, HIF-1α, CTGF, and COL-1 (mainly including COL-1A1 and COL-1A2) (Figure 7). There was a stepwise interaction among NF-κB, HIF-1α, CTGF, and COL-1A1 or COL-1A2.

## 4 Discussion

This study was conducted to mainly evaluate the function of left ventricular systolic function *in vivo*, observe histological manifestations, and initially explore the putative mechanisms, and assessed the protective effects of PDTC treatment on RIHD at an early stage. First, we found that there was no significant difference in left ventricular systolic function at the 14th day after irradiation. Second, the histopathological results displayed myocardial cell edema, myocardial cell-disordered arrangement, abnormal cell infiltration, and pro-fibrotic cardiac change in irradiated hearts. Third, enhanced levels of P50, P65, HIF-1α, CTGF, and COL-1 occurred when challenged by irradiation. Finally, the most important finding was that PDTC treatment, to some extent, alleviated cardiac damage at a very early stage in locally irradiated hearts, which might be attributed to suppress NF-κB.

Our data showed that there was no significant impairment of the left ventricular systolic function *in vivo* at the 14th day after irradiation in rats, consistent with previous studies also showed that no change in cardiac function occurred 4 months after radiation, and the deterioration of heart function occurred 4–6 months after radiation (Boerma et al., 2008; Mezzaroma et al., 2012). Our previous finding also indicated that the basic left ventricular function of isolated rat hearts was also not impaired at the 14th day post-radiation (Wang et al., 2016). The heart had its own reserve capacity, and it might keep a relative normal function at a compensatory stage in a short time after adverse stress. Echocardiography, an assessment of cardiac function *in vivo*, was a non-invasive and repeatable examination. Compared with the previous detection of isolated cardiac function, echocardiography data could be similar to the performance of patients who experienced thoracic radiotherapy.

Left ventricular systolic function might not be a very sensitive index of cardiac injury at an early stage due to the cardiac reserve capacity and compensatory ability. In other words, cardiac damage might occur without the impairment of the cardiac systolic function. Therefore, we euthanized the rats and performed HE and Masson’s trichrome staining to check the cardiac morphological changes. HE staining was mainly used to observe the morphology and arrangement of cardiomyocytes. Masson’s trichrome staining was a common method to assess fibrosis in the hearts with collagen being stained green. Previous studies indicated that rats were exposed to local heart irradiation with a single dose of 18, 20, or 24 Gy and were observed for 3–6 months; alterations myocardial inflammatory infiltration and interstitial fibrosis were progressed in time-dependent and dose-dependent manners (Sridharan et al., 2012; Gürses et al., 2014). Yi et al. (2020) indicated that cardiac tissue presented disordered arrangement of myocardial cells at the 21st day after irradiation in mice. In the present study, we found disordered arrangement of myocardial cells and abnormal cell infiltration at the 14th day after irradiation, suggesting that a much earlier change of myocardial cells might have occurred. As for which kind of cell infiltration, immunofluorescence or immunohistochemistry with specific markers could be used. In parallel, we also found the onset of cardiac fibrosis based on the results of Masson’s trichrome staining and the expression of COL-1 at the 14th day post-irradiation. Above all, PDTC might alleviate myocardial cell-disordered arrangement, abnormal cell infiltration, and fibrotic change induced by radiation. Up to now, it was the first study to find that PDTC had a protective effect on radiation-induced heart damage at an early stage.

The mechanism of RIHD at an early stage was not well understood, and there were several hypotheses according to preclinical evidence. Activation of inflammatory response or oxidative stress and fibrosis has been reported (Schultz-Hector and Trott 2007; Tapio 2016; Ping et al., 2020). NF-κB is an inducer of inflammation. Weintraub and Halle revealed that NF-κB increased in the irradiated vessel areas (Halle et al., 2010; Weintraub et al., 2010). In the present study, the local heart of the rat model was irradiated, and both mRNA and protein expression of cardiac tissue were detected. As a result, NF-κB was activated. Combined with the aforementioned disordered arrangement of myocardial cells or abnormal cell infiltration and fibrosis, we speculated that perhaps there was a relationship between the disordered arrangement of myocardial cells and fibrosis. After the PPI network analysis, we hypothesized that HIF-1α and CTGF were the bridge between NF-κB and COL-1. Van Uden indicated that NF-κB directly regulated the expression of HIF-1α under the normal oxygen condition (Van Uden et al., 2008). Additionally, HIF-1α could regulate the secretion of fibrogenic cytokine CTGF, promote the synthesis of collagen type I and fibronectin, and increase the proliferation of fibroblast (Angelini et al., 2015). Preliminarily, we observed an increase in the protein and mRNA levels of NF-κB, HIF-1α, and protein of COL-1 after irradiation to the local heart in a rat model. The levels of CTGF mRNA and protein only showed a trend of attenuation by PDTC but did not reach statistical significance. One possible explanation is that there are extremely complex interactions between HIF-1α and CTGF. Haydont observed that TGF-β1 directly activated the transcription of the CTGF gene after mesenchymal cells were exposed to irradiation (Haydont et al., 2008). Vozenin-Brotons indicated that the lower concentration of TGF-β1 sustained the expression of CTGF in intestinal radiation fibrosis (Vozenin-Brotons et al., 2003). Thus, we deduced that only suppressing NF-κB might be inadequate to interfere with the transcription and expression of CTGF. Meanwhile, the results of Western blot revealed that radiation-induced COL-1 increased by irradiation, but the mRNA level of COL-1 did not reach statistical significance. Theoretically, transcription of mRNA was prior to the translation of protein, but the processes of transcription and translation had their own half-lives and occurred at different times. At the end point of the present study (at the 14th day post-radiation), transcription of COL-1 mRNA was low, but the translation of COL-1 protein was high. This finding provided a new insight into RIHD at a very early stage. Confident study on the NF-κB/HIF-1α/CTGF/COL-1 signaling pathway was regulated by the activation of NF-κB in rat myocardial cells or primary cultures *in vitro* experiments or transgenic animals was needed.

PDTC is an antioxidant and an inhibitor of NF-κB. PDTC played an anti-inflammatory role, which could interfere with the production of pro-inflammatory cytokines and inhibit the activation of NF-κB (Cuzzocrea et al., 2002). Practically, PDTC has been examined for its preclinical safety evaluation in the liver, brain, nerves, and fat tissues and has reached the standard of safety application (Chabicovsky et al., 2010). In a rat model with hypertension and myocardial ischemia, PDTC has been identified to inhibit cardiac inflammatory response, relieve ventricular remodeling, maintain normal tissue structure, and protect cardiac function (Theuer et al., 2002; Cau et al., 2015). In the present study, PDTC treatment might alleviate structural damage in the locally irradiated heart. Such a protective effect might be associated with the suppression of NF-κB. Taken together, these results suggested that the suppression of NF-κB, HIF-1α, CTGF, and COL-1 might be a potential mechanism for the cardiac protection of PDTC. Furthermore, due to the advantage of safe, easy application, and low cost, PDTC might be a promising agent for the clinical therapy of RIHD.

There are several limitations to be mentioned herein. First, we observed the effects of PDTC on left ventricular systolic function and cardiac inflammation and pro-fibrotic cardiac change only at the 14th day after irradiation. Therefore, long-term studies, especially up to 3–6 months, are needed. Second, we observed primary cardiac injury in rats by HE staining did not evaluate the pro-inflammatory cell infiltrate and the kind of cells are involved with specific markers. Third, we showed that NF-κB, HIF-1α, CTGF, and COL-1 might be related to the early stage of radiation-induced cardiac injury. PPI network analysis indicated that there might be a stepwise interaction between NF-κB, HIF-1α, CTGF, and COL-1A1 or COL-1A2; however, confident study that signaling expression regulated by the activation of the NF-κB/HIF-1α/CTGF/COL-1 signaling pathway regulated by the activation of NF-κB in rat myocardial cells or primary cultures *in vitro* experiments or transgenic animals was needed to be conducted.

## 5 Conclusion

In conclusion, this study demonstrated that radiation-induced myocardial cell edema, partial myocardial cell-disordered arrangement, inflammation infiltration, and pro-fibrotic cardiac change could be observed as early as the 14 days after irradiation in rats, but there was no significant damage to the function of left ventricular at the very early stage. PDTC treatment to some extent alleviated structural damage in the locally irradiated heart. Such a protective effect might be associated with the suppression of NF-κB. This study provides evidence that PDTC might be a potential clinical approach to address RIHD.

## Data Availability

The raw data supporting the conclusions of this article will be made available by the authors, without undue reservation.
